# Long-term topical cyclosporin A therapy in Thygeson's superficial punctate keratitis: a case report

**DOI:** 10.1186/1757-1626-1-415

**Published:** 2008-12-23

**Authors:** Murat Hasanreisoglu, Rahamim Avisar

**Affiliations:** 1 Department of Ophthalmology, Rabin Medical Center, Petach Tikva and Sackler Faculty of Medicine, Tel Aviv University, Tel Aviv, Israel

## Abstract

**Purpose:**

To describe a patient with Thygeson's superficial punctate keratitis successfully treated with long-term cyclosporin A.

**Case presentation:**

A 15-year-old boy presented with long-term ocular symptoms of foreign body sensation, burning, irritation, tearing, and transient photophobia. One year's treatment with steroidal agents had led to only partial improvement. Clinical and laboratory evaluation yielded a diagnosis of Thygeson's superficial punctate keratitis. Treatment with 0.5% topical cyclosporin A for the last 4.5 years has been associated with symptom resolution and corneal clearing, without side effects.

**Conclusion:**

Topical cyclosporin A seems to effective and safe for the treatment of Thygeson's superficial punctuate keratitis and should be considered in selective cases when topical steroids fail or in there is a high risk of complications from long-term steroid treatment. Considering the chronicity of Thygeson's superficial punctate keratitis, further prospective studies of long-term cyclosporine A treatment are needed.

## Introduction

In 1950, Thygeson was the first to describe the clinical entity of transient, bilateral, coarse corneal epithelial opacities, without associated stromal involvement or corneal edema, which was later termed Thygeson's superficial punctate keratitis (TSPK) [1]. TSPK usually occurs in the second to third decade of life, though patients of all ages can be affected. It has a chronic course characterized by exacerbations and remissions. The duration of the disease ranges from 1 month to 24 years; some cases of a longer course of up to 41 years have been described as well [2].

The management of TSPK varies with disease severity. During quiescent periods with minimal irritation, simple lubricating drops or no treatment is sufficient [3]. During acute exacerbations, topical corticosteroids have been found to decrease signs and symptoms [1]. Therapeutic contact lenses can be used to reduce the irritation in more symptomatic patients [3].

We describe a patient with TSPK that responded only partially to long-term corticosteroids. A switch to topical cyclosporin A was associated with very favorable results, without any side effects

## Case presentation

**Figure 1:**
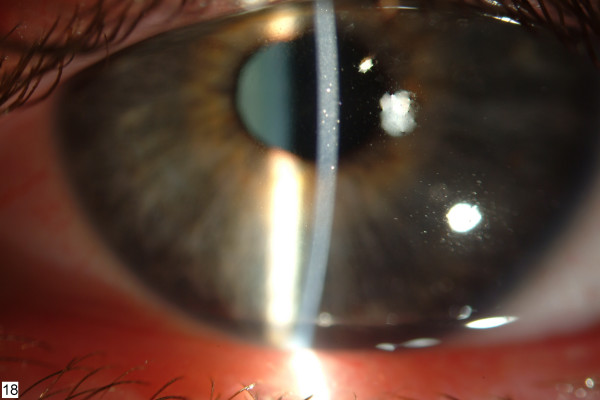
**Left cornea before cyclosporin A treatment**. Note the slightly elevated, grayish-white subepithelial opacities. Clinical appearance was similar in the right eye.

A 15-year-old otherwise healthy boy presented to the Cornea Service at Rabin Medical Center complaining of foreign body sensation, burning, tearing, and irritation in both eyes accompanied by mild transient photophobia. One year's treatment with topical steroids and various antibiotics had been associated with only partial remission with intermittent exacerbations. Clinical examination showed a best-corrected visual acuity (BCVA) of 6/9 OU with correction. Intraocular pressure was 15 mmHg in both eyes. No pathologic findings were noted in the eyelids. There was only trace conjunctival injection. On slit-lamp examination, discrete, centrally located, slightly elevated, grayish-white corneal subepithelial opacities were detected, with a granular texture bilaterally (Figure 1). They did not stain with fluorescent dye. The anterior chambers and vitreous were clear, with no inflammatory reaction, cells, flare or any kind of opacification. A diagnosis of TSPK was made on the basis of the clinical appearance of bilateral superficial punctuate epithelial keratopathy with minimal conjunctival reaction, the chronicity of the disease with remissions and exacerbations, and the partial response to topical corticosteroids. The patient did not agree to use soft contact lenses, and he was already receiving topical corticosteroids for a long period. Therefore, we decided on a trial with topical cyclosporin A. After receiving consent from the patient and his family, the patient was prescribed 0.5% cyclosporin A drops in castor oil 3 times a day.

Over the next 3 months, there was a slow but noticeable improvement in clinical symptoms. On follow-up slit-lamp examination, the corneal subepithelial opacities had cleared almost completely, and the minimal conjunctival injection had disappeared. Continued treatment for the next 4 years led to total regression of the disease. Every attempt to taper the cyclosporin A treatment resulted in a reappearance of symptoms and of corneal opacities.

**Figure 2:**
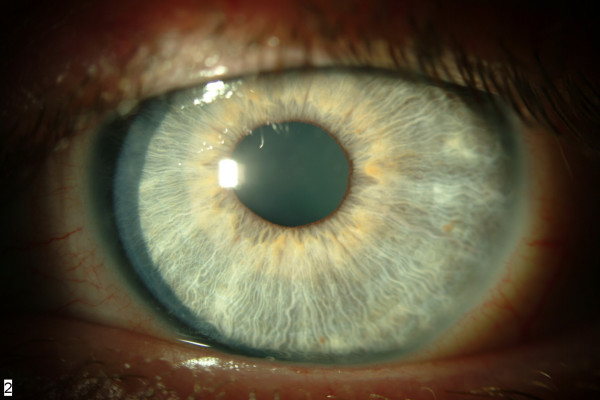
**Left cornea after cyclosporin A treatment**. Note the clear cornea, with full resolution of the opacities with no sequelae. The clinical appearance was similar in right eye.

At the most recent follow-up visit, 4.5 years after presentation, the patient was still applying cyclosporin A drops 3 times daily. He had no symptoms. Visual acuity was 6/6 in both eyes. There was no conjunctival inflammation, and the corneas were clear without opacification. (Figure 2) Endothelial cell count was within normal range in both eyes.

## Discussion

We describe a patient with TSPK treated with topical cyclosporin A drops for 4.5 years with excellent results. To our knowledge, this is the longest course of topical cyclosporin A treatment for TSPK reported in the literature.

Cyclosporin A is a potent immunomodulator that was reported to be helpful as a primary or adjunctive therapy in the treatment of keratoconjunctivitis sicca, vernal conjunctivitis, atopic conjunctivitis, and superior limbic keratoconjunctivitis [4]. When administered systemically, it may be associated with such adverse effects as acute and chronic nephrotoxicity, neurotoxicity, hypertension, and new-onset diabetes [5]. However, in accordance with the low cyclosporin A concentrations found in blood and in tissues other than those at the ocular surface, no systemic adverse effects were associated with topical ophthalmic cyclosporin A treatment [6]. In phase 3 clinical trials of 0.05% and 0.1% ophthalmic emulsions of cyclosporin for the treatment of dry eye disease, the most common treatment-related adverse events were burning eye, stinging eye, and conjunctival hyperemia. The safety profile of cyclosporin treatment was excellent [7,8].

The effectiveness and safety of cyclosporin specifically for the treatment of TSPK has been tested so far in two prospective studies. Both showed that 2% cyclosporin A treatment led to suppression of the epithelial and subepithelial opacities and satisfactory control of the condition. The authors recommended it as a safe alternative to corticosteroids [9,10].

Topical corticosteroids remain the mainstay of treatment for TSPK because of their widespread availability and effectiveness. However, their long-term use is known to lead to side effects. Furthermore, one study found that prolongation of the course of TPSK may be secondary to the introduction of corticosteroids [2]. Therefore, considering the relatively young age of onset of TPSK and the consequent need for prolonged therapy, together with the apparent effectiveness and minor, if any, side effects of cyclosporin A, we suggest that topical cyclosporin A can be used as first-line therapy in selective cases of Thygeson's superficial punctuate keratitis when topical steroids fail or in the presence of a high risk of complications from long-term steroid treatment. Further prospective studies of long-term cyclosporin A treatment in this setting are needed.

## Consent

Written informed consent was obtained from the patient for publication of this case report. A copy of the written consent is available for review by the Editor-in-Chief of this journal.

## Competing interests

The authors declare that they have no competing interests.

## Authors' contributions

MH and RA were integrally involved in the patient's management and diagnosis. Both also greatly contributed to writing of the case report. MH performed the literature search and final copy-editing of the paper. All authors read and approved the final manuscript.
